# Prognostic value of the HALP score in patients with testicular cancer: a retrospective study

**DOI:** 10.1007/s12094-025-03973-3

**Published:** 2025-06-17

**Authors:** Ahmet Burak Agaoglu, Atike Pınar Erdogan, Mustafa Sahbazlar, Ferhat Ekinci

**Affiliations:** Division of Medical Oncology, Faculty of Medicine, Celal Bayar University, Yunusemre, Manisa, Turkey

**Keywords:** Testicular cancer, HALP score, Prognosis, Survival, Inflammation

## Abstract

**Purpose:**

The hemoglobin, albumin, lymphocyte, and platelet (HALP) score is a novel immune nutritional index associated with the prognosis of various malignancies. This study aimed to evaluate the clinical relevance of the HALP score in predicting recurrence, metastasis, and survival outcomes in patients with testicular cancer.

**Methods:**

This retrospective study included 131 patients with histologically confirmed testicular cancer who were treated between January 2010 and December 2024. HALP scores were calculated using the baseline laboratory parameters. An optimal cutoff value of 304.56 was identified using receiver operating characteristic (ROC) curve analysis (AUC: 0.820; 95% CI 0.708–0.902; *p* < 0.001). Patients were stratified into high and low HALP groups based on this threshold value. Survival outcomes were evaluated using Kaplan–Meier, and Cox proportional hazards regression analyses.

**Results:**

The median HALP score was 531.1 (IQR: 348.8–728.5). A low HALP score (≤ 304.56) was significantly associated with poorer 1-year overall survival (54.5% vs. 98.2%, *p* < 0.001) and a 15.6-fold increased risk of death (HR 15.604, 95% CI 2.718–89.645, p = 0.001) based on Cox regression analysis. In the multivariate analysis, a low HALP score (HR: 7.684, *p* = 0.016), the presence of comorbidity (HR 13.528, *p* = 0.002), and tunica albuginea invasion (HR 7.255, *p* = 0.030) were identified as independent predictors of recurrence or metastasis. In addition, the HALP score was significantly associated with disease stage (*p* < 0.001), with lower scores more commonly observed in patients with advanced-stage disease.

**Conclusions:**

The HALP score was a simple, inexpensive, and effective prognostic biomarker in testicular cancer. A score ≤ 304.56 is independently associated with a higher risk of recurrence, metastasis, and mortality. Incorporating HALP into routine assessments may improve risk stratification and clinical decision-making in this patient population.

## Introduction

Testicular cancer is the most common malignancy in men aged 15–40 years, and its incidence has been gradually increasing worldwide over the past decades [1]. Although early-stage disease is often curable with surgery and/or chemotherapy, a subset of patients may still experience recurrence or progression, underscoring the need for reliable prognostic tools [2]. The current risk stratification largely relies on the histopathological staging and serum tumor markers, such as alpha-fetoprotein (AFP), beta-human chorionic gonadotropin (beta-hCG), and lactate dehydrogenase (LDH). However, these markers do not fully capture host-related factors, such as immune and nutritional status, which are increasingly recognized as significant modulators of cancer behavior [3, 4].

The hemoglobin, albumin, lymphocyte, and platelet (HALP) score is a novel composite biomarker that reflects systemic inflammation and nutritional reserve. Prognostic relevance has been noted in malignancies of the colon, stomach, kidneys, and urinary bladder [5–7]. The HALP score is simple to calculate using routine laboratory parameters and has shown promise in identifying patients with a higher risk of poor outcomes. The recent evidence has also highlighted the prognostic importance of nutritional status in patients with cancer; being underweight is significantly correlated with poor outcomes [8]. These findings underscore the potential value of integrating nutritional-inflammatory indices, such as the HALP score, into clinical evaluation and risk stratification models.

Although a few studies have investigated the use of the HALP score in patients with testicular cancer, its clinical application in this context remains insufficiently defined [9, 10]. Accordingly, we investigated the role of the HALP score in testicular cancer, focusing on its association with survival outcomes and recurrence or metastasis risk.

Systemic inflammation and nutritional status are increasingly recognized as important factors in cancer progression and its clinical outcomes. The HALP score is a novel composite biomarker that reflects both the inflammatory response and nutritional status. It has demonstrated utility in various malignancies, including gastrointestinal and genitourinary cancers [5–7]. Although a few studies have investigated the use of the HALP score in patients with testicular cancer, its clinical applicability in this context remains uncertain and lacks consensus [8, 9].

Given the need for reliable and easily accessible prognostic tools for management of testicular cancer, we aimed to evaluate the clinical relevance of the HALP score in this patient population. Specifically, we investigated the association of this signature with survival outcomes and disease recurrence in a cohort of patients with testicular cancer.

## Methods

This retrospective study included 131 patients diagnosed with testicular cancer who were followed up and treated at our institution between 2010 and 2024. Clinical, pathological, and laboratory data were obtained from hospital records. Patients with incomplete clinical or laboratory information or those whose treatment or follow-up continued in other institutions and thus could not be reliably evaluated were excluded. Only patients with sufficiently detailed and complete data were included in the final analysis (*n* = 131). Patients aged < 18 years, those with insufficient medical documentation, and those with follow-up shorter than 6 months were excluded from the analysis. We adopted a complete case analysis approach without performing any data imputation. Additional exclusion criteria were the presence of a second malignancy, hematologic diseases, acute infections, chronic inflammatory conditions, or autoimmune disorders. Tumor staging followed the TNM classification outlined in the 8th edition by the American Joint Committee on Cancer.

Demographic characteristics (age, BMI, Eastern Cooperative Oncology Group performance status (ECOG-PS), and comorbidities), tumor-related variables (tumor size, stage, histologic subtype, lymphovascular invasion, rete testis, spermatic cord, epididymis, and tunica albuginea invasion), and treatment data (type of chemotherapy and number of BEP cycles) were collected. In addition, recurrence and metastasis status, metastasis sites, and preoperative and postoperative tumor markers (AFP, β-hCG, and LDH) were evaluated. Routine laboratory parameters—including serum calcium, creatinine, estimated glomerular filtration rate (eGFR), ALT, ALP, total and direct bilirubin, neutrophil count, hemoglobin, albumin, lymphocyte, and platelet counts—were recorded at baseline. The HALP score was calculated using the following formula [11].

HALP score = Hemoglobin (g/L) × Albumin (g/L) × Lymphocyte count (/L) ÷ Platelet count (/L).

The optimal cutoff value of the HALP score for predicting recurrence or metastasis was determined using receiver operating characteristic (ROC) curve analysis. The cutoff point (304.56) was selected based on the Youden index, which maximizes the sum of the sensitivity and specificity. However, no internal validation (e.g., bootstrapping) was conducted. Patients were then categorized into two groups based on this cutoff value (≤ 304.56 vs. > 304.56).

Analyses were conducted using SPSS 15.0 (Chicago, IL, USA). Categorical variables are expressed as counts and percentages, and continuous variables are reported as medians with interquartile ranges (IQR). Survival probabilities were estimated using the Kaplan–Meier method, and differences across groups were tested using the log-rank test. To identify predictors of recurrence, metastasis, and mortality, univariate Cox regression models were first applied, followed by multivariate analyses incorporating variables with *p* < 0.05. The final multivariate model was developed using a backward stepwise elimination procedure. A two-tailed *p* value of < 0.05 was considered statistically significant across all analyses.

The study received ethical approval from the Manisa Celal Bayar University Ethics Committee (Decision No: E-20478486–050.04–968551; dated 05 March 2025) and was conducted by the ethical standards of the Declaration of Helsinki.

## Results

A total of 131 patients with testicular cancer were retrospectively analyzed. The median age was 32 (IQR: 25–40), and the majority of patients (84%) had an ECOG-PS of 0. Most of the tumors were stage I (60%) and histologically classified as nonseminomatous germ cell tumors (58.5%). Metastasis was detected in 23.7% of the patients, and recurrence was observed in 8.3% of the patients (Table 1).

**Table 1:** Clinicopathological characteristics

Age, median (IQR)	32 (25–40)
ECOG-PS	
0, *n* (%)	111 (84%)
1, *n* (%)	17 (13%)
2, *n* (%)	3 (2.3%)
Comorbidity	
No, *n* (%)	120 (91.6%)
Yes, *n* (%)	11 (8.5%)
Tumor size, median (IQR)	4.5 (3.1–96.5)
Stage	
1, *n* (%)	78 (60%)
2, *n* (%)	24 (18.5%)
3, *n* (%)	28 (21.5%)
Laterality	
Left, *n* (%)	62 (47.3%)
Right, *n* (%)	69 (52.7%)
Lymphovascular invasion	
No, *n* (%)	80 (64.5%)
Yes, *n* (%)	44 (35.5%)
Rete testis invasion	
No, *n* (%)	65 (52.8%)
Yes, *n* (%)	58 (47.2%)
Epididymal invasion	
No, *n* (%)	108 (87.1%)
Yes, *n* (%)	16 (12.9%)
Spermatic cord invasion	
No, *n* (%)	117 (92.1%)
Yes, *n* (%)	10 (7.9%)
Tunica albuginea invasion	
No, *n* (%)	109 (86.5%)
Yes, n (%)	17 (13.5%)
Histologic subtype	
Seminoma, *n* (%)	54 (41.5%)
Non-seminoma, *n* (%)	76 (58.5%)
Metastasis status	
No, *n* (%)	100 (76.3%)
Yes, *n* (%)	31 (23.7%)
Recurrence	
No, *n* (%)	111 (84.7%)
Yes, *n* (%)	10 (8.3%)
Treatment decision	
Follow-up, *n* (%)	58 (44.3%)
Chemotherapy, *n* (%)	73 (55.7%)
Number of adjuvant BEP cycles, median (IQR)	3 (1–4)
Death	
No, *n* (%)	120 (91.6%)
Yes, *n* (%)	11 (8.4%)

Baseline laboratory parameters, including AFP, β-hCG, LDH, hemoglobin, albumin, lymphocyte, and platelet counts, are presented in Table 2.

**Table 2:** Laboratory values

	Median (IQR)
Preop AFP, (ng/mL)	3.8 (2.15–104)
Preop β-hCG, (mIU/mL)	3.3 (0.96–45)
Preop LDH, (U/L)	270.5 (195.8–462.8)
Postop AFP, (ng/mL)	2.9 (2–8.9)
Postop β-hCG, (mIU/mL)	0.5 (0.25–2)
Postop LDH, (U/L)	197 (168–245)
Serum albumin, (g/dL)	4.5 (4.1–4.75)
Serum calcium, (mg/dL)	9.6 (9.3–9.9)
Glomerular filtration rate, (mL/min/1.73 m^2^)	116.5 (99.8–127)
Serum creatinine, (mg/dL)	0.82 (0.7–0.9)
Alanine aminotransferase, (U/L)	21 (14–28)
Total bilirubin, (mg/dL)	0.55 (0.3–0.74)
ALP, (U/L)	77 (69–100)
Hemoglobin, (g/dL)	15.1 (14.2–15.8)
Platelet count, (/μL × 10^3^)	247 (199.8–283)
Neutrophil count, (/μL)	5650 (4285–6925)
Lymphocyte count, (/μL)	1945 (1480–2500)
HALP Score	531.1 (348.8–728.5)

*Preop* preoperative, *Postop* postoperative, *HALP* hemoglobin × albumin × lymphocyte/platelet.

The median HALP score was 531.1 (IQR: 348.8–728.5). The optimal cutoff value for the HALP score was identified as 304.56 based on ROC curve analysis (AUC: 0.820; 95% CI 0.708–0.902; *p* < 0.001), with a sensitivity of 71.4% and specificity of 90.2%. Based on this threshold, 110 patients (83.9%) had high HALP scores and 21 patients (16.1%) had low scores.

**Fig. 1:**
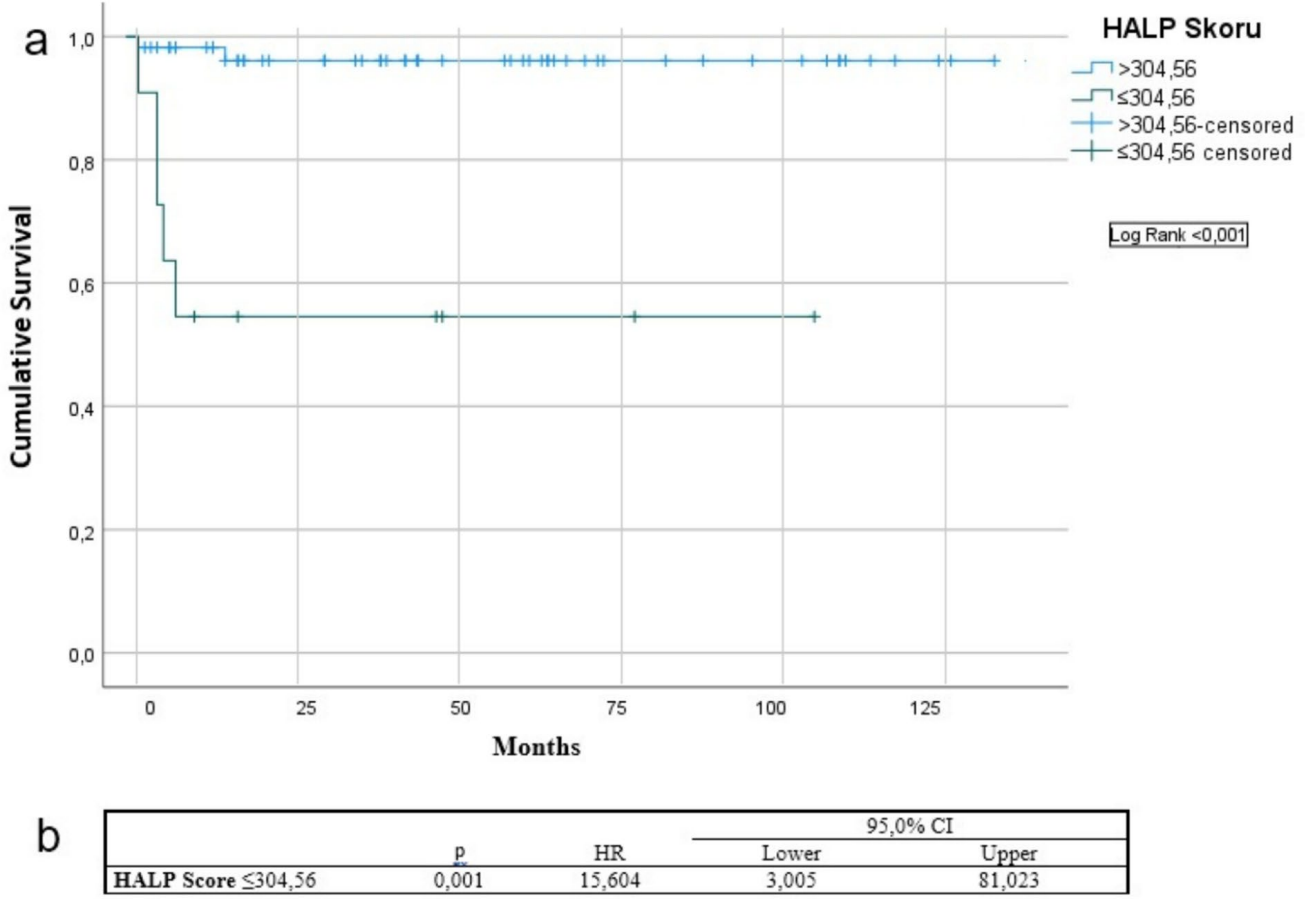
Kaplan–Meier plot showing 1-year overall survival stratified by HALP score (≤ 304.56 vs. > 304.56) **a**. Cox regression results presenting the hazard ratio, p value, and 95% confidence interval for low HALP score as a predictor of overall survival **b**

Patients were categorized according to this cutoff value. Kaplan–Meier survival analysis revealed significantly lower overall survival in patients with HALP ≤ 304.56 compared to those with higher scores (1-year OS: 54.5% vs. 98.2%; log-rank, *p* < 0.001). As shown in Fig. 1, univariate analysis showed a 15.6-fold elevated risk of death in patients with low HALP scores (HR 15.604; 95% CI 3.005–81.023; *p* = 0.001).

In multivariate Cox regression analysis, a low HALP score (HR: 7.684, *p* = 0.016), the presence of comorbidity (HR: 13.528, *p* = 0.002), and tunica albuginea invasion (HR: 7.255, *p* = 0.030) were identified as independent predictors of recurrence or metastasis. These associations suggest that the HALP score reflects not only systemic inflammatory and nutritional status but also correlates with biologically aggressive disease (Table 3).

**Table 3:** Univariate Cox regression analysis of risk factors for recurrence and metastasis

	Univariate		Multivariate	
	HR (lower–upper 95% CI)	*P* value	HR (lower–upper 95% CI)	*P* value
Age (≥ 32 vs < 32)	1.038 (1.008–1.069)	0.012*		
BMI (≥ 25 vs < 25)	0.948 (0.856–1.049)	0.298		
ECOG-PS (≥ 2 vs < 2)	1.529 (0.197–11.841)	0.684		
Comorbidity (yes vs no)	4.004 (1.307–12.269)	0.015*	13.528 (2.504–73.078)	0.002*
Stage (2 vs 1)	2.486 (0.756–8.175)	0.134		
Stage (3 vs 1)	8.560 (2.931–25.00)	< 0.001*		
Laterality (left vs right)	1.325 (0.541–3.243)	0.538		
LVI (yes vs no)	4.732 (1.619–13.831)	0.005*		
RTI (yes vs no)	5.159 (1.467–18.140)	0.011*	6.343 (0.748–53.790)	0.090
EI (yes vs no)	5.317 (1.988–14.220)	0.001*		
SCI (yes vs no)	4.794 (1.322–17.388)	0.017*		
TAI (yes vs no)	3.720 (1.406–9.844)	0.008*	7.255 (1.209–43.539)	0.030*
Histologic subtype (seminoma vs non-seminoma)	0.903 (0.366–2.229)	0.824		
HALP score (≤ 304.56 vs ˃304.56)	3.681 (1.224–11.072)	0.020*	7.684 (1.454–40.610)	0.016*
Treatment decision (follow-up vs chemotherapy)	1.247 (0.506–3.070)	0.631		
Number of BEP cycles	1.921 (1.077–3.427)	0.027*		
Diagnosis-to-chemotherapy interval	1.009 (0.995–1.024)	0.195		

*LVI* lymphovascular invasion, *RTI* rete testis invasion, *EI* epididymal invasion, *SCI* spermatic cord invasion, *TAI* tunica albuginea invasion, *BEP* bleomycin–etoposide–cisplatin, *HALP* hemoglobin × albumin × lymphocyte/platelet,

*Statistically significant at *p* < 0.05.

In addition, the HALP score was significantly associated with disease stage (*p* < 0.001), with lower scores more commonly observed in patients with advanced-stage disease. No significant associations were observed between the HALP score and histologic subtype or tumor laterality (Table 4).

**Table 4:** Association between different clinicopathological parameters and HALP score

Parameters	HALP Score		
	High	Low	*P* value
ECOG-PS			
< 2, *n* (%)	55 (83.3)	11 (17.7)	1.000
≥ 2, *n* (%)	2 (100)	0 (0)	
Tumor size			
< 4, *n* (%)	25 (92.5)	2 (7.5)	0.178
≥ 4, *n* (%)	31 (77.5)	9 (22.5)	
Stage			
1, *n* (%)	35 (97.2)	1 (2.8)	< 0.001*
2, *n* (%)	12 (92.4)	1 (7.6)	
3, *n* (%)	10 (55.6)	8 (44.4)	
Laterality			
Left, *n* (%)	27 (81.8)	6 (18.1)	0.663
Right, *n* (%)	30 (85.8)	5 (14.2)	
LVI			
No, *n* (%)	36 (90)	4 (10)	0.165
Yes, *n* (%)	19 (76)	6 (24)	
Rete testis invasion			
No, *n* (%)	29 (87.9)	4 (12.1)	0.511
Yes, *n* (%)	26 (81.3)	6 (18.7)	
Epididymal invasion			
No, *n* (%)	46 (85.2)	8 (14.8)	0.406
Yes, *n* (%)	9 (75)	3 (25)	
SCI			
No, *n* (%)	51 (83.6)	10 (16.4)	1.000
Yes, *n* (%)	5 (83.4)	1 (16.6)	
Histologic subtype			
Seminoma, *n* (%)	25 (89.3)	3 (10.7)	0.505
Nonseminoma, *n* (%)	32 (80)	8 (20)	

*LVI* lymphovascular invasion, *SCI* spermatic cord invasion, *HALP* hemoglobin × albumin × lymphocyte/platelet

*Statistically significant at *p* < 0.05

## Discussion

To our knowledge, this is the first retrospective study to evaluate the prognostic value of the HALP score in testicular cancer using a ROC-derived optimal cutoff. This offers novel insights into its potential clinical applicability in a young patient population with high expected cure rates.

Our findings demonstrate that a low HALP score is significantly associated with poor 1-year overall survival and serves as an independent predictor of recurrence or metastasis. The HALP score, a composite index based on the hemoglobin, albumin, lymphocytes, and platelets, may reflect both nutritional reserves and systemic inflammatory burden, which are known to influence cancer outcomes.

These results are consistent with those of recent studies exploring the role of immune nutritional markers in testicular cancer. Bumbasirevic et al. reported that lower HALP and prognostic nutritional index (PNI) scores were associated with higher clinical stage and larger tumor size in patients with testicular germ cell tumors [9]. Similarly, Ekici et al. found that low HALP scores correlated with increased tumor aggressiveness and worse outcomes [10]. These findings reinforce the relevance of immuno-nutritional assessment in testicular cancer, where young patients may appear clinically well despite harboring a biologically aggressive disease.

Although several inflammation- and nutrition-based indices, such as the neutrophil-to-lymphocyte ratio (NLR), platelet-to-lymphocyte ratio (PLR), systemic immune-inflammation index (SII), and controlling nutritional status (CONUT) score, have been evaluated across various cancers, their application in testicular cancer remains limited and heterogeneous. In contrast, the HALP score integrates key hematologic and nutritional parameters into a single metric, offering a more comprehensive and stable reflection of the host status. A recent meta-analysis by Xu et al. confirmed HALP’s superiority of HALP over other composite markers in predicting overall and recurrence-free survival across malignancies [6]. Further comparative studies are needed to establish the optimal prognostic index for testicular cancer.

Recent evidence underscores the intricate interplay between systemic inflammation, nutritional status, and cancer progression [12]. Chronic inflammation can promote tumorigenesis by inducing genomic instability, enhancing angiogenesis, and enabling metastatic dissemination [13]. Conversely, poor nutritional status, often a consequence of the catabolic state induced by cancer, can impair immune competence, reduce chemotherapy tolerance, and worsen oncologic outcomes [14–16]. Biologically, the HALP score captures the key components of cancer-related malnutrition and immune dysregulation. Low albumin and hemoglobin levels reflect protein-energy malnutrition and reduced oxygen-carrying capacity, while lymphopenia and thrombocytosis are linked to immune suppression and tumor-promoting inflammation [17–20]. Thus, HALP may serve not only as a prognostic marker but also as a proxy for cancer-related cachexia and frailty.

Multivariate Cox regression analysis revealed that the HALP score, comorbidities, and tunica albuginea invasion were independent predictors of recurrence or metastasis. Notably, the HALP score demonstrated a hazard ratio of 7.684 (*p* = 0.016), underscoring its prognostic strength beyond that of traditional histopathologic factors. Importantly, this association persisted even after adjusting for tumor stage and comorbidities, reinforcing the potential utility of the HALP score in enhancing risk stratification beyond the conventional TNM classification. These findings align with emerging evidence supporting the role of immune nutritional indices in cancer prognosis. Previous studies have associated low HALP scores with a higher tumor burden and worse survival outcomes in renal, pancreatic, and gynecologic cancers [5–7, 21, 22].

In oncological practice, malnourished patients often exhibit reduced tolerance to cytotoxic chemotherapy, resulting in dose reductions or delays that may compromise the efficacy of the treatment. Studies have shown that an impaired immune nutritional status correlates with a higher incidence of adverse events, prolonged hospital stays, and suboptimal clinical outcomes [23]. It is plausible that similar mechanisms underlie the poor prognosis observed in our testicular cancer patients with low HALP scores, as these individuals may be less capable of withstanding the rigors of BEP-based treatment regimens.

This study had several limitations. First, the sample size (*n* = 131) was relatively modest, particularly for the multivariate analysis. To mitigate potential bias, only patients with complete and reliable clinical and laboratory data were included, while those with missing data or who were followed up externally were excluded. Sensitivity analyses excluding borderline or uncertain cases yielded consistent results, supporting the robustness of our findings, despite this limitation. Nevertheless, future studies with larger, prospectively enrolled cohorts across multiple centers are required to validate the generalizability of our findings.

Second, although the HALP cutoff value (304.56) was derived via ROC analysis using the Youden index, internal validation techniques such as bootstrapping were not employed in this study. This may raise concerns regarding overfitting, particularly given the limited cohort size. Future research should include such validation strategies to strengthen the confidence in the proposed cutoff.

Third, the interpretation of comorbidity is limited by the small number (*n* = 11) and heterogeneity of comorbid conditions. The high hazard ratio observed (HR = 13.528) could be partially explained by residual confounding, small sample bias, and differences in treatment tolerance. Some of these patients may have died from noncancer causes, further complicating the interpretation.

Fourth, although the 1-year survival difference between the HALP groups was significant, long-term follow-up data were not uniformly available across the cohort. Although survival appeared stable after the first year, the recent diagnoses limited the extended tracking. Thus, long-term outcomes could not be reliably assessed, warranting further prospective studies to confirm these findings.

Finally, although we assessed treatment-related variables, such as the time to treatment initiation, we believe that a more detailed evaluation of these factors is needed. Future studies should incorporate comprehensive treatment data to better account for potential confounding factors.

In summary, the HALP score appears to be a promising immune nutritional biomarker that independently predicts the prognosis of patients with testicular cancer. Its simplicity, objectivity, and integration of multiple host-related factors support its potential role in clinical risk stratification. However, it should currently be considered a complementary prognostic tool rather than the sole basis for treatment decisions. Further prospective studies are needed to validate its clinical utility and define how it can be best integrated into standardized care pathways.

## Conclusion

Our findings suggest that the HALP score is an independent prognostic marker in testicular cancer, associated with recurrence, metastasis, and mortality. However, due to the retrospective and single-center nature of this study, its role should be interpreted cautiously. At present, HALP may be used as a supportive risk-stratification tool alongside established clinical parameters. Prospective multicenter studies with standardized follow-ups are needed to confirm its clinical utility and integration into treatment algorithms. In clinical workflows, HALP could serve as an early alert for patients at higher risk who may benefit from closer surveillance or nutritional interventions, particularly when traditional staging is insufficient to capture host vulnerability.

## Data Availability

The data that support the findings of this study are available from the corresponding author upon reasonable request.
